# Supplementary material for: The inequality labor loss risk from future urban warming and adaptation strategies
Source: Nat Commun. 2022 Jul 6;13:3847. doi: 10.1038/s41467-022-31145-2 (PMC9259578; doi:10.1038/s41467-022-31145-2)
Supplement: Supplementary file 1 — Supplementary Information [file 41467_2022_31145_MOESM1_ESM.docx]

**Supplementary Information**

**Supplementary Methods**

**WRF/UCM model setting**

First, according to related research focused at a similar scale^1^, we selected some important physical attributes and model options for the WRF, which are detailed in Supplementary Table 3.

In terms of the initial boundary conditions under the RCP 6.0 scenario, we used the global bias-corrected dataset outputs from NCAR’s Community Earth System Model, which participated in phase 5 of the Coupled Model Intercomparison Project (CMIP5) (https://rda.ucar.edu/datasets/ds316.1/index.html/) and supported the Intergovernmental Panel on Climate Change Fifth Assessment Report (IPCC AR5). This dataset contains all the variables needed in this study for simulations with the WRF model, which has been widely used in previous studies about future urban climate under different climate change assumptions^1, 2^.

For the baseline scenario, we selected the NCEP Final (FNL) Operational Global Analysis data as the initial boundary conditions. These data are on 1 degree by 1 degree grids prepared operationally every six hours. This product is from the Global Data Assimilation System and other sources (https://rda.ucar.edu/datasets/ds083.2/).

The spatial patterns of contemporary urban areas were obtained from the Chinese Academy of Sciences Resource and Environmental Science Data Center (http://www.resdc.cn/).

To divide the urban grids into three types, we treated urban grids with populations greater than 1 million as commercial centers, those with populations greater than 100 thousand and less than 1 million as high-intensity residential areas, and the remaining urban grids as low-intensity residential areas, according to the population and urban area size data for first-tier cities in China according to Chinese government^3^.

Other important physical attributes that we modified for the urban canopy model are detailed in Supplementary Table 4.

For all the simulations, we set the same modeling domain to include portions of the Pacific Ocean, Mongolia and the Korean Peninsula and all areas of China at 20 km resolution, as shown in Supplementary Fig.5. To consider all changes occurring across all cities and to avoid the influence of nonurban areas, we selected all 20 × 20-km grids with urban land use fractions higher than 10% as urban grids, as 76% of all urban residents were located in these grids.

To spin up the model, each modeling period began one week before each studied period.

Details on the calibration of the WRF results from the baseline data against the observed air temperature in the 10 cities are provided in Supplementary Fig.7 and Supplementary Fig.8. Overall, the results demonstrate the ability of the WRF to reproduce the climate of the baseline scenario.

**WBGT calculation method**

According to the related research^4^, WBGT in shade conditions at stable wind speed (1 m/s) was calculated as:

$WBGT=0.33T_{a}+0.67T_{pwb}$ (1)

where *T_a_* represents the 2-m air temperature (°C) and *T_pwb_* represents the 2-m psychometric wet bulb temperature. Based on related research^1^, we calculated *T_pwb_* as:

$T_{pwb}=\frac{\alpha T_{a}+\beta T_{de}}{\alpha+\beta}$ (2)

in which

$\alpha=0.00066*ps$ (3)

$\beta=\frac{4098*P}{{(T_{de}+237.8)}^{2}}$ (4)

where *T_de_* represents the dewpoint temperature (°C), *ps* represents barometric surface pressure (kPa) and *P* represents the vapor pressure (kPa).

*T_de_* was calculated as:

$T_{de}=\frac{243\ln\left( \frac{P}{0.6112} \right)}{17.67}-ln(\frac{P}{0.6112})$ (5)

**The cost of applying green roof**

Here, we assume that it takes n years ($Y_{n}$) to recover all the investment costs ($I_{costs}$) of adopting green roof under our green roof scenario, so the following formula can be established:

$${Re}_{loss}\times Y_{n}={Area}_{green\_roof}\times I_{costs}$$

${Re}_{loss}$ represent the annually recovered economic losses from the implementation of green roof, which is 212.63 million US dollars according to our estimation (Table.s3). According to the recommendation from Beijing government^5^, the standard of $I_{costs}$ is 310 CNY/m^2^ for the extensive green roof system^5^. ${Area}_{green\_roof}$ represents the total area of green roof, according to our settings in the WRF/UCM, it can be calculated as:

$${Area}_{green roof}=\sum_{i=1}^{j} {Ugrid}_{i}\times{Furb}_{i}\times{Fbuild}_{i}\times\frac{R_{roof}}{R_{roof}+R_{road}}\times80\%$$

Where, *j* represents the total number of urban grids, ${Ugrid}_{i}$ is the total area of the urban grid *i,* ${Furb}_{i}$ is the urban land-use fraction in grid *i,* ${Fbuild}_{i}$ is the building plan fraction for specific urban land-use type (Table.s4), $R_{roof}$ is the road width and $R_{road}$ is the roof width.

**Supplementary Figures**


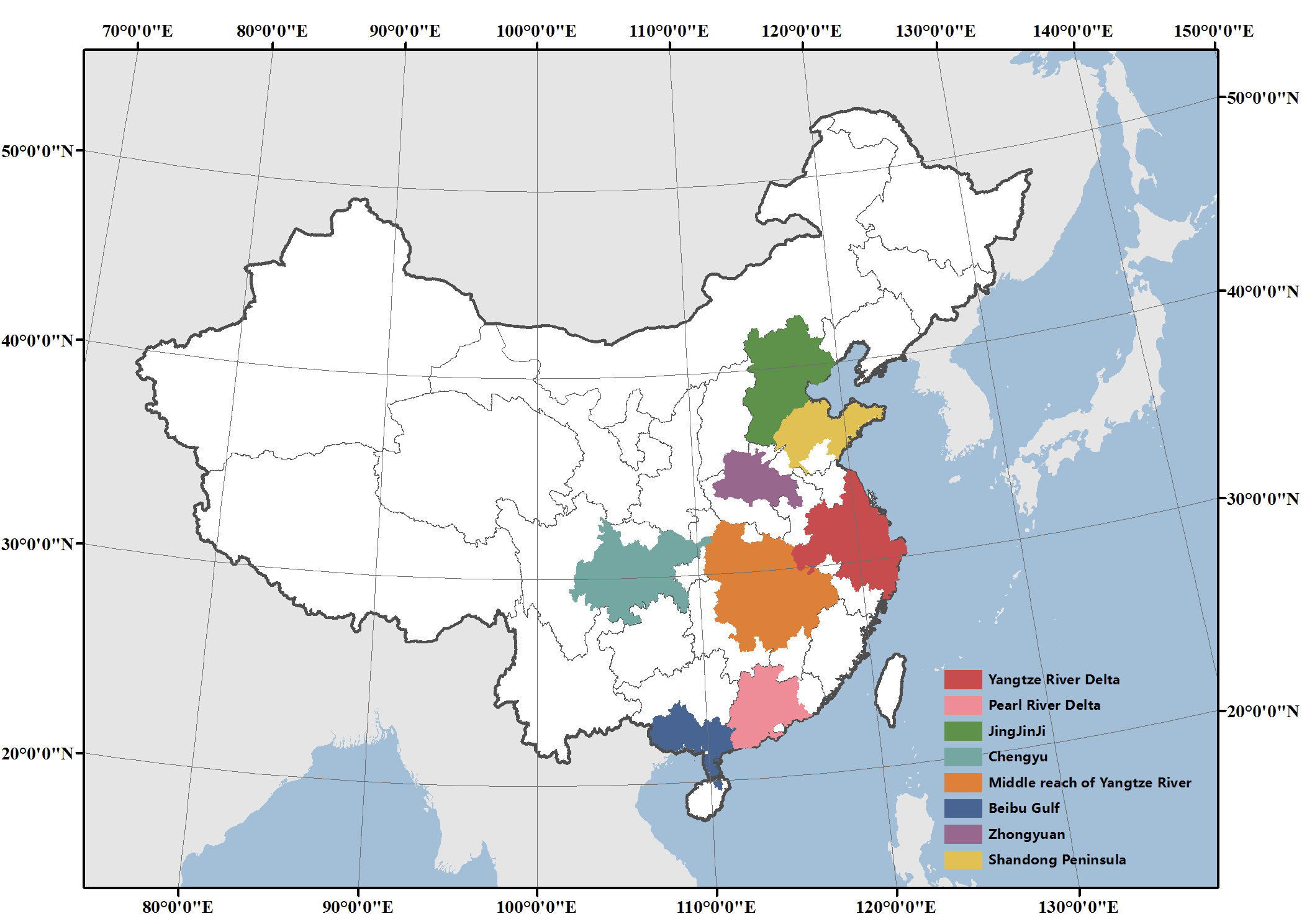


**Supplementary Fig.1:** **The boundary of the 8 urban agglomerations that we selected for this study**


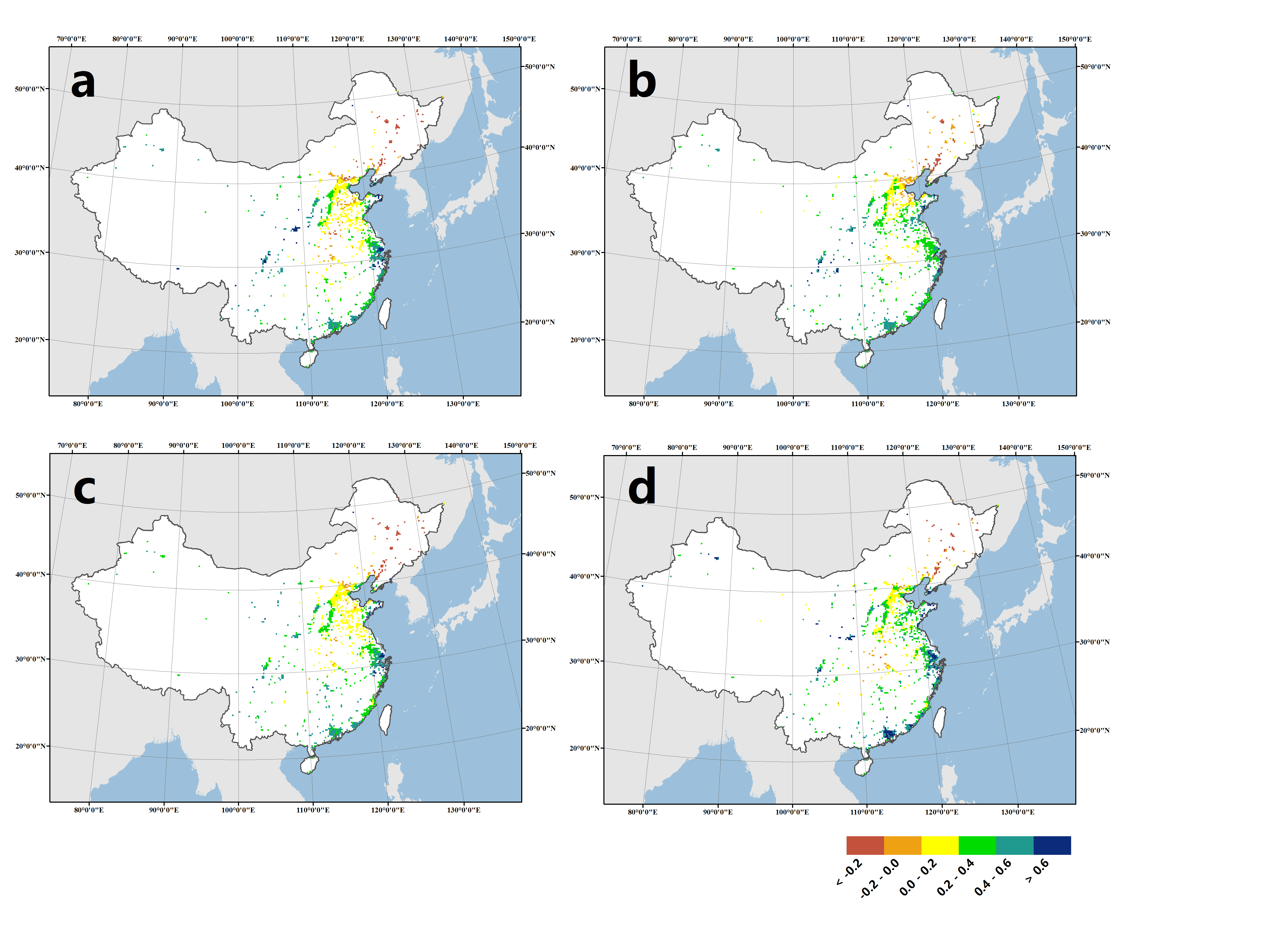


**Supplementary Fig.2:** **Reduction in the average wet-bulb global temperature in shadow (WBGTs) during the hottest part of the day in the hottest month resulting from several cooling adaptation strategies, including (a) green roofs, (b) cool walls, (c) cool ground surfaces and (d) full adaptation.** Areas in white are areas in which the urban land use fraction is projected to be less than 10% in the future.

**
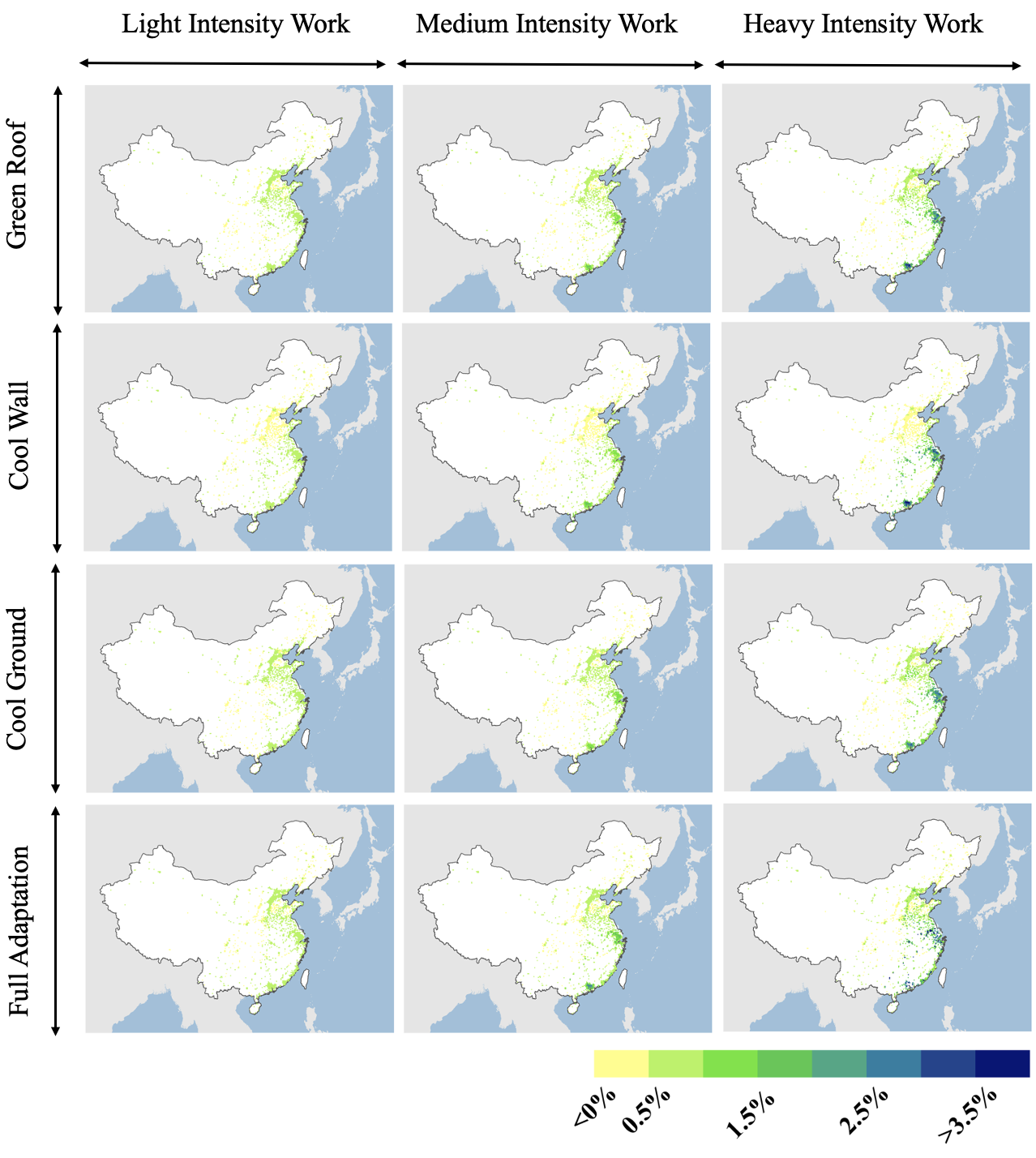
**

**Supplementary Fig.3: Average recovered percentage of worker productivity for three types of work due to application of the three adaptation strategies.** The exposure-response function developed by Kjellstrom^6^ was used to estimate the heat impacts on hourly worker productivity during all work hours in summer. For the calculation methods, please see the methods section.


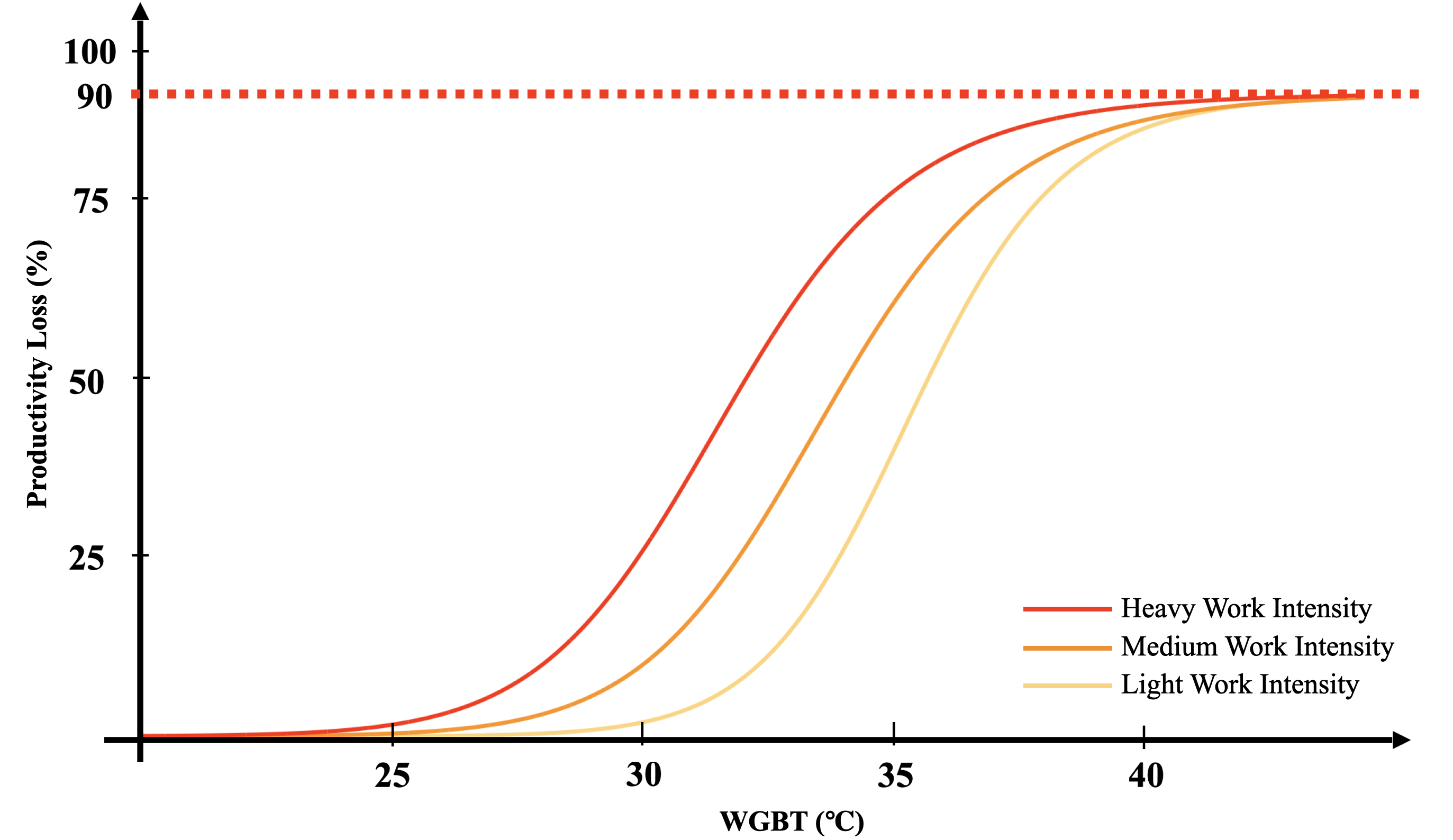


**Supplementary Fig.4: The exposure-response functions describing the relationship between WGBT and the ratio of labor loss for three different work intensities.** The impacts on productivity were measured on an hourly basis in related field studies^7^.


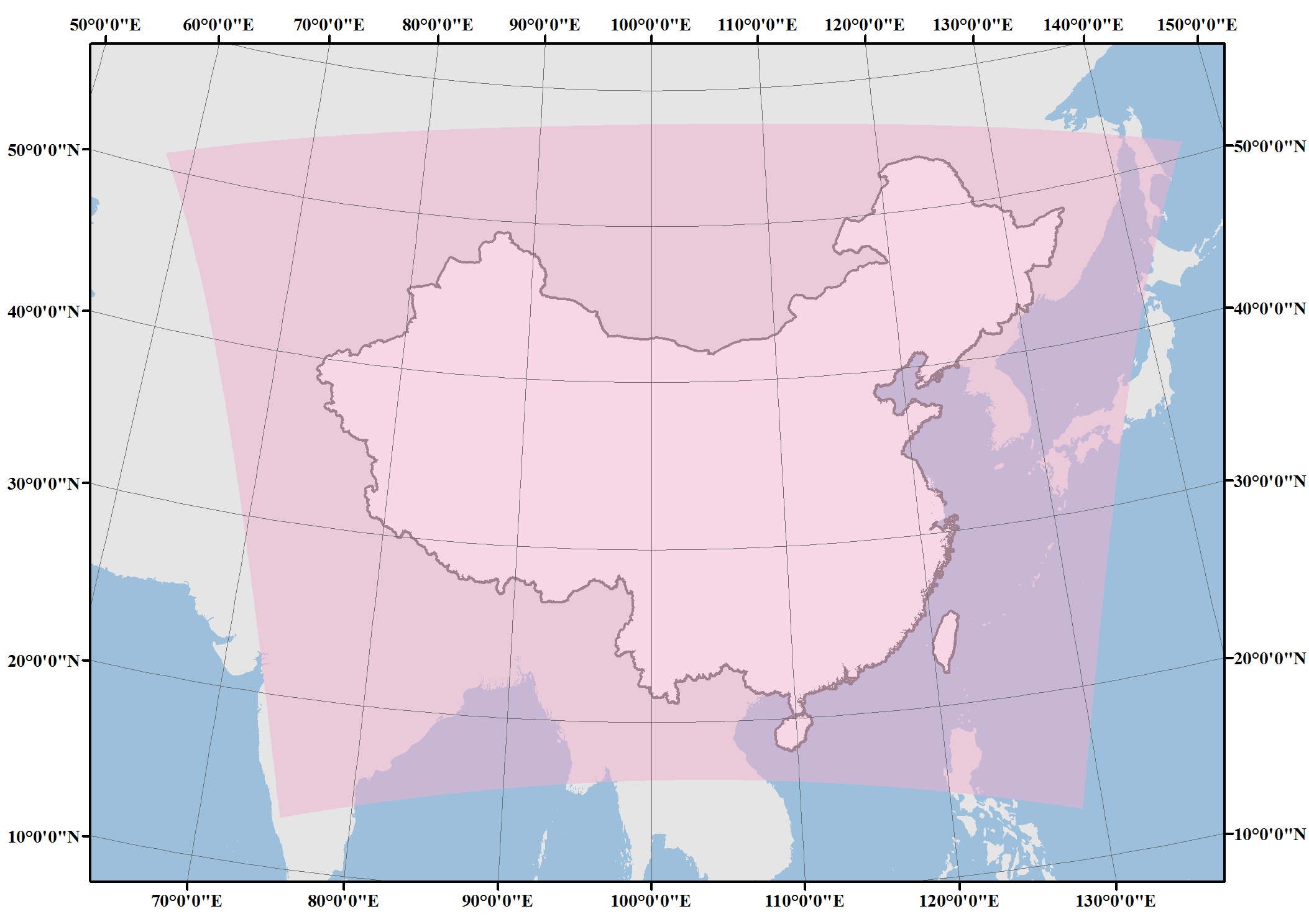


**Supplementary Fig.5: Domain of the WRF simulation at 20-km resolution.**


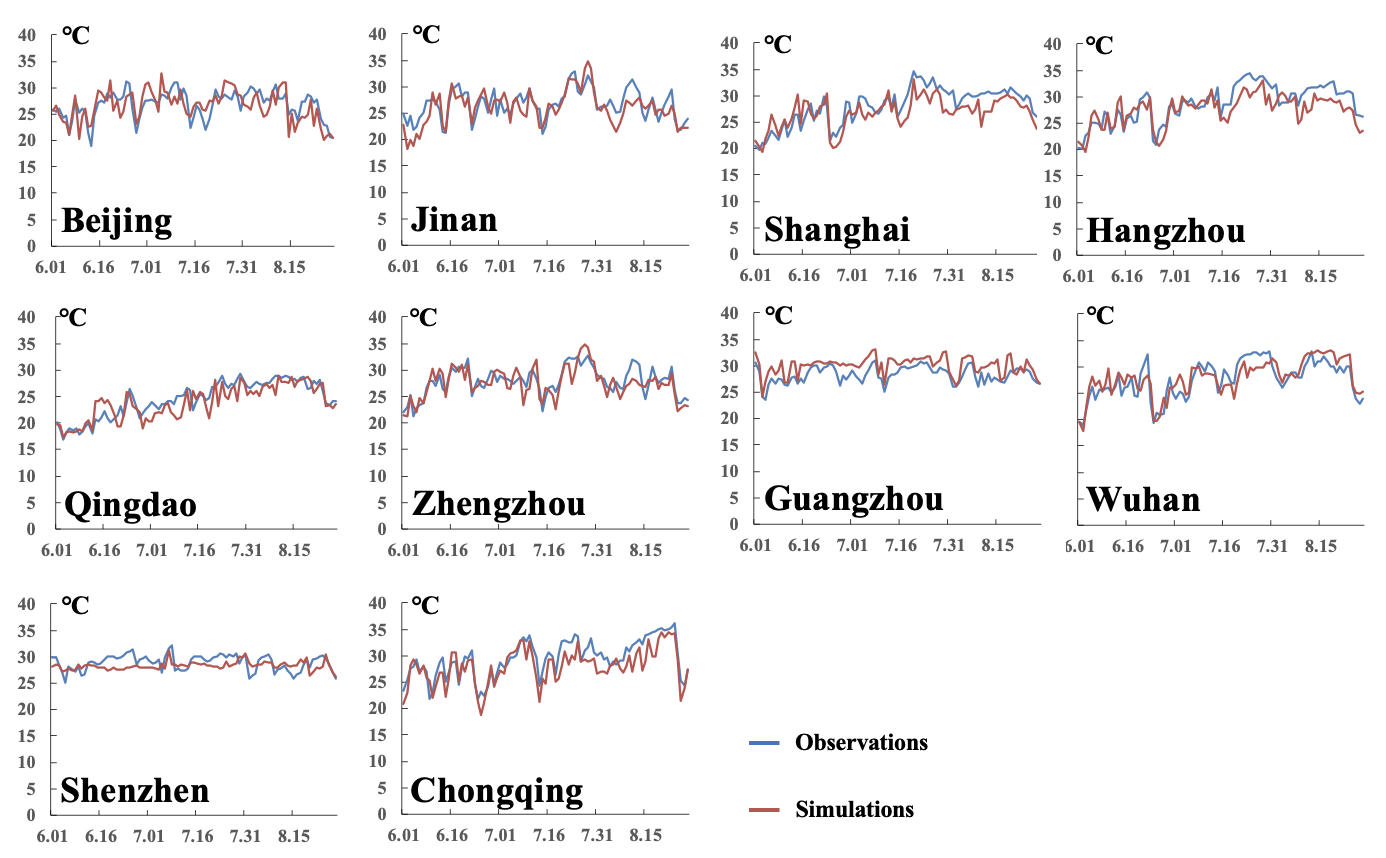


**Supplementary Fig.6: The distributions of daily 2-m air temperature between the WRF simulation output and observation data of 10 urban meteorological observatories from 1 June to 31 August 2015.** The observation data were obtained from the National Earth System Science Data Center (http://www.geodata.cn). For comparison, we processed the point observation data into the nearest WRF simulation grid with 20-km resolution.


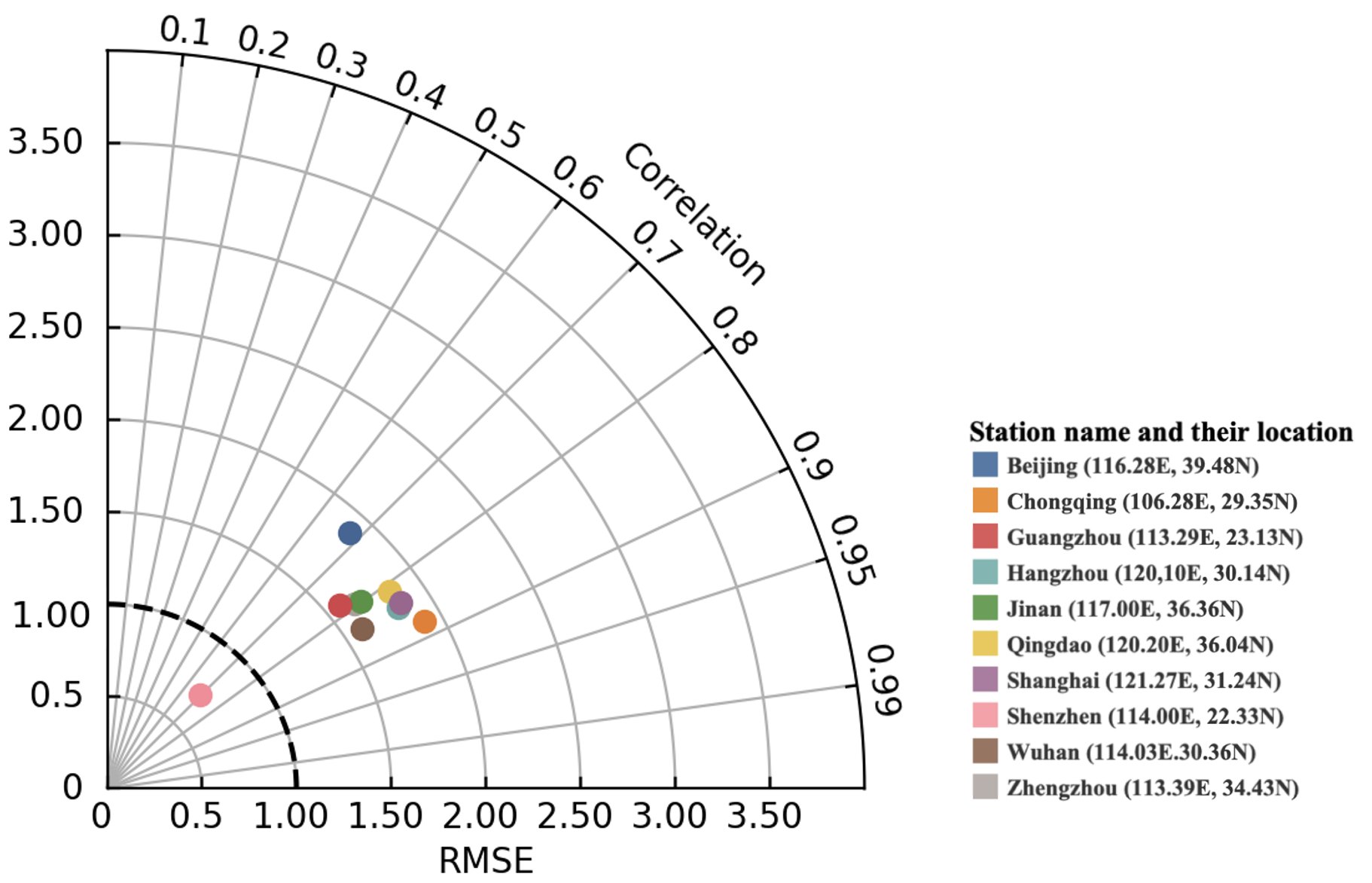


**Supplementary Fig.7: Comparison of the distributions of daily 2-m air temperature between the WRF simulation output and observation data of** **10 urban meteorological observatories from 1 June 2015 to 31 August 2015.** Each dot denotes one city. The correlation coefficients between the WRF outputs and the observational field pass the significance test (p<0.05) for each of the 10 urban meteorological observatories, and the standard deviations and RMSE of two series are within 0.5-2.5, indicating that our model is able to depict the temperature situation under the baseline scenario and to simulate climate change under future scenarios.

**Supplementary Tables**

**Supplementary Table 1. Annual economic losses from reduced labor due to extreme warming in different sectors under baseline and future scenarios (units: million US dollars).** We only account for the data from all urban grids of all possible working hours (08:00–20:00) in summer.

|  | **Baseline (2010-2020)**  **Future** | | | | **RCP6.0 (2050-2060)** | | | | **RCP8.5 (2050-2060)** | | | |
| --- | --- | --- | --- | --- | --- | --- | --- | --- | --- | --- | --- | --- |
|  | **Total** | **M&T*** | **Construction** | **Services** | **Total** | **M&T** | **Construction** | **Services** | **Total** | **M&T** | **Construction** | **Services** |
| **YRD** | 1013.76 | 111.85 | 881.50 | 20.40 | 1990.79 | 241.49 | 1692.68 | 56.62 | 2378.18 | 283.83 | 2032.93 | 61.42 |
| **PRD** | 382.82 | 97.46 | 270.88 | 14.49 | 518.28 | 132.77 | 365.50 | 20.01 | 628.86 | 161.44 | 442.87 | 24.55 |
| **JJJ** | 299.04 | 63.36 | 221.55 | 14.12 | 581.15 | 140.16 | 394.90 | 46.09 | 786.13 | 177.65 | 558.72 | 49.76 |
| **CY** | 113.99 | 26.39 | 82.03 | 5.57 | 202.94 | 49.81 | 139.92 | 13.21 | 216.68 | 52.33 | 151.35 | 12.99 |
| **MYR** | 188.65 | 45.29 | 133.82 | 9.54 | 474.37 | 120.96 | 323.52 | 29.89 | 474.16 | 117.87 | 328.23 | 28.06 |
| **BG** | 59.89 | 9.29 | 47.11 | 3.50 | 80.21 | 13.24 | 61.69 | 5.27 | 80.76 | 13.26 | 62.20 | 5.30 |
| **ZY** | 7.22 | 2.33 | 4.48 | 0.42 | 347.77 | 133.41 | 182.29 | 32.07 | 360.17 | 131.67 | 198.91 | 29.59 |
| **SD** | 118.47 | 38.45 | 73.46 | 6.56 | 212.73 | 73.24 | 120.53 | 18.97 | 267.80 | 90.39 | 155.75 | 21.65 |
| **ALL** | 2569.58 | 509.70 | 1967.13 | 92.75 | 5110.00 | 1087.13 | 3760.95 | 261.92 | 5815.13 | 1199.24 | 4357.25 | 258.64 |

* Manufacturing and transportation

**Supplementary Table 2.** **The annually recovered economic losses from reacquired labor support due to cooling from the implementation of infrastructure-related urban adaptation strategies under RCP6.0 scenario** (**units: million US dollars**).

|  | **Green Roof** | **Cool Wall** | **Cool Ground** | **Full Adaptation** |
| --- | --- | --- | --- | --- |
| **All** | 212.63 | 259.89 | 191.80 | 265.73 |
| **YRD** | 160.13 | 194.92 | 183.49 | 198.04 |
| **PRD** | 20.82 | 33.95 | 26.29 | 65.04 |
| **JJJ** | 46.18 | 42.77 | 28.07 | 40.96 |
| **CY** | 9.37 | 7.61 | 1.17 | 10.26 |
| **MYR** | 38.86 | 30.29 | 7.54 | 32.06 |
| **BG** | 3.13 | 6.52 | 3.06 | 7.54 |
| **SD** | 51.59 | 55.68 | 41.04 | 27.14 |
| **ZY** | 11.71 | 8.40 | 3.56 | 2.61 |

**Supplementary Table 3. WRF physics options.**

| **Item** | **Option** | **Reference** |
| --- | --- | --- |
| Microphysics | Purdue Lin microphysics scheme | Lin et al.,^8^ |
| Longwave radiation | RRTM scheme | Mlawer et al. ^9^ |
| Shortwave radiation | Dudhia | Dudia ^10^ |
| Land surface | Noah land surface model | Chen and Dudhia ^11^ |
| Surface layer | MM5/Monin-Obukhov | Janjic ^12^ |
| Cumulus parameterization | Grell 3-D | Grell and Devenyi ^13^ |

**Supplementary Table 4. WRF-UCM input parameters.**

| Parameter | Units | Low-intensity  Residential | High-intensity  Residential | Commercial  Center |
| --- | --- | --- | --- | --- |
| Building plan fraction | - | 0.33 | 0.50 | 0.55 |
| Anthropogenic heat | Wm^-2^ | 5 | 15 | 75 |
| Canyon height-to-width ratio | - | 0.25 | 0.5 | 2.0 |
| Mean building height | m | 5 | 5 | 20 |

**Reference**

1. Krayenhoff E Scott, Moustaoui Mohamed, Broadbent Ashley M, Gupta Vishesh, Georgescu Matei. Diurnal interaction between urban expansion, climate change and adaptation in US cities. *Nature Climate Change* **8**, 1097 (2018).

2. Broadbent Ashley Mark, Krayenhoff Eric Scott, Georgescu Matei. The motley drivers of heat and cold exposure in 21st century US cities. *Proceedings of the National Academy of Sciences* **117**, 21108-21117 (2020).

3. Prc The State Council of The. About Adjusting the Criteria for Urban Size Division. (ed^(eds) (2014).

4. Andrews Oliver, Le Quéré Corinne, Kjellstrom Tord, Lemke Bruno, Haines Andy. Implications for workability and survivability in populations exposed to extreme heat under climate change: a modelling study. *The Lancet Planetary Health* **2**, e540-e547 (2018).

5. Chen Xi, Shuai Chenyang, Chen Zhenhao, Zhang Yu. What are the root causes hindering the implementation of green roofs in urban China? *Science of the Total Environment* **654**, 742-750 (2019).

6. Kjellstrom Tord, Freyberg Chris, Lemke Bruno, Otto Matthias, Briggs David. Estimating population heat exposure and impacts on working people in conjunction with climate change. *International Journal of Biometeorology* **62**, 291-306 (2018).

7. Bröde Peter, Fiala Dusan, Lemke Bruno, Kjellstrom Tord. Estimated work ability in warm outdoor environments depends on the chosen heat stress assessment metric. *International Journal of Biometeorology* **62**, 331-345 (2018).

8. Lin Yuh-Lang, Farley Richard D., Orville Harold D. Bulk parameterization of the snow field in a cloud model. *Journal of Applied Meteorology and Climatology* **22**, 1065-1092 (1983).

9. Mlawer Eli J, Taubman Steven J, Brown Patrick D, Iacono Michael J, Clough Shepard A. Radiative transfer for inhomogeneous atmospheres: RRTM, a validated correlated‐k model for the longwave. *Journal of Geophysical Research: Atmospheres* **102**, 16663-16682 (1997).

10. Dudhia Jimy. Numerical study of convection observed during the winter monsoon experiment using a mesoscale two-dimensional model. *Journal of Atmospheric Sciences* **46**, 3077-3107 (1989).

11. Chen Fei, Dudhia Jimy. Coupling an advanced land surface–hydrology model with the Penn State–NCAR MM5 modeling system. Part I: Model implementation and sensitivity. *Monthly Weather Review* **129**, 569-585 (2001).

12. Janjić Zaviša I. The step-mountain coordinate: Physical package. *Monthly Weather Review* **118**, 1429-1443 (1990).

13. Grell Georg A, Dévényi Dezső. A generalized approach to parameterizing convection combining ensemble and data assimilation techniques. *Geophysical Research Letters* **29**, 38-31-38-34 (2002).
