# Supplementary material for: The inequality labor loss risk from future urban warming and adaptation strategies
Source: Nat Commun. 2022 Jul 6;13:3847. doi: 10.1038/s41467-022-31145-2 (PMC9259578; doi:10.1038/s41467-022-31145-2)
Supplement: Supplementary file 2 — Reporting Summary [file 41467_2022_31145_MOESM2_ESM.pdf]

nature portfolio

Corresponding author(s): Weichun Ma, Haidong Kan, Patrick L. Kinney, NCOMMS-21-263678

Last updated by author(s): May 1, 2022

Reporting Summary

Nature Portfolio wishes to improve the reproducibility of the work that we publish. This form provides structure for consistency and transparency in reporting. For further information on Nature Portfolio policies, see our Editorial Policies and the Editorial Policy Checklist.

Statistics

For all statistical analyses, confirm that the following items are present in the figure legend, table legend, main text, or Methods section.

n/a	Confirmed	
☐	☒	The exact sample size (*n*) for each experimental group/condition, given as a discrete number and unit of measurement
☐	☒	A statement on whether measurements were taken from distinct samples or whether the same sample was measured repeatedly
☐	☒	The statistical test(s) used AND whether they are one- or two-sided<br>*Only common tests should be described solely by name; describe more complex techniques in the Methods section.*
☒	☐	A description of all covariates tested
☐	☒	A description of any assumptions or corrections, such as tests of normality and adjustment for multiple comparisons
☐	☒	A full description of the statistical parameters including central tendency (e.g. means) or other basic estimates (e.g. regression coefficient) AND variation (e.g. standard deviation) or associated estimates of uncertainty (e.g. confidence intervals)
☐	☒	For null hypothesis testing, the test statistic (e.g. *F*, *t*, *r*) with confidence intervals, effect sizes, degrees of freedom and *P* value noted<br>*Give P values as exact values whenever suitable.*
☒	☐	For Bayesian analysis, information on the choice of priors and Markov chain Monte Carlo settings
☒	☐	For hierarchical and complex designs, identification of the appropriate level for tests and full reporting of outcomes
☐	☒	Estimates of effect sizes (e.g. Cohen's *d*, Pearson's *r*), indicating how they were calculated

Our web collection on statistics for biologists contains articles on many of the points above.

Software and code

Policy information about availability of computer code

Data collection	We applied Version 3.9.1 the Advanced Research Weather Research and Forecasting (WRF) model coupled to a single-layer urban canopy scheme to dynamically downscale different climate change scenarios across the mainland China.
Data analysis	All data analysis were performed and plotted on the Python platform (Version 3.8.10) using the open-source libraries, including: Pandas (Version 3.8.10), Numpy (Version 1.20.3), Xarray (Version 0.19.0), and Matplotlib (Version 3.4.3).

For manuscripts utilizing custom algorithms or software that are central to the research but not yet described in published literature, software must be made available to editors and reviewers. We strongly encourage code deposition in a community repository (e.g. GitHub). See the Nature Portfolio guidelines for submitting code & software for further information.

Data

Policy information about availability of data

All manuscripts must include a data availability statement. This statement should provide the following information, where applicable:

- Accession codes, unique identifiers, or web links for publicly available datasets
- A description of any restrictions on data availability
- For clinical datasets or third party data, please ensure that the statement adheres to our policy

All the initial boundary conditions data used in this study can be downloaded from The Research Data Archive managed by the National Center for Atmospheric Research at https://rda.ucar.edu/. Other relevant data in this study are available from the corresponding authors upon reasonable request

Field-specific reporting

Please select the one below that is the best fit for your research. If you are not sure, read the appropriate sections before making your selection.

- [ ] Life sciences
- [ ] Behavioural & social sciences
- [x] Ecological, evolutionary & environmental sciences

For a reference copy of the document with all sections, see nature.com/documents/nr-reporting-summary-flat.pdf

Ecological, evolutionary & environmental sciences study design

All studies must disclose on these points even when the disclosure is negative.

Study description	With the use of a regional climate model, we aimed to quantify the possible influences of the interactions among climate change and potential urban adaptation strategies (installation of green roofs, cool walls, and cool ground surfaces) on future urban warming. Based on a widely used ERF, we assessed the economic costs to urban residents attributable to urban heat condition.
Research sample	This study involved 231 cities out of a total of 333 Chinese cities and 196 million urban residents out of a total of 643 million urban residents. These cities were selected based on the criterion of an urban land area above a certain size (10 km2). The spatial patterns of contemporary urban population distribution were obtained from the NCAR Integrated Assessment Modeling Project (https://www.cgd.ucar.edu/iam/). The spatial distribution of urban areas were obtained from the Chinese Academy of Sciences Resource and Environmental Science Data Center (http://www.resdc.cn/).
Sampling strategy	These cities were selected based on the criterion of an urban land area above a certain size (10 km2) , Because when the total area of the city exceeds more than 10 km2, the thermal environment of the city could be significantly higher than that of the suburbs.
Data collection	For the climate model, in regard to the initial boundary conditions under the RCP 6.0 scenario, we used global bias-corrected dataset outputs retrieved from the National Center for Atmospheric Research (NCAR) community earth system model. For the baseline scenario, we selected the NCEP Final (FNL) Operational Global Analysis data as the initial boundary conditions.<br>For the data on city-specific ratios of the average working population and unemployed population, proportion of the working population employed in each sector and hourly wages in each sector were obtain ed from the China City Statistical Yearbook (2010-2019).<br>The spatial patterns of contemporary urban population distribution were obtained from the NCAR Integrated Assessment Modeling Project (https://www.cgd.ucar.edu/iam/). The spatial distribution of urban areas were obtained from the Chinese Academy of Sciences Resource and Environmental Science Data Center (http://www.resdc.cn/).<br>All these data were collected from related website by Cheng He.
Timing and spatial scale	We attempted to quantify the risk of summer heat pressure by combining climate modeling over the baseline period (i.e., the recent past [June 1 to August 31 in each year from 2010 to 2020]) with climate projections for the middle of this century ([june 1 to August 31 in each year from 2050 to 2060]) based on climate change models at 20 km spatial resolution with existing population projections.
Data exclusions	No data were excluded.
Reproducibility	We ran 2 variations of the same task and replicated the model ouput for baseline results.
Randomization	This is bot relevant to our study, as we modeled all changes under different scenarios.
Blinding	Blinding is not possible, as this study is mainly based on climate model, the results of the models have been verified.

Did the study involve field work? [ ] Yes [x] No

Reporting for specific materials, systems and methods

We require information from authors about some types of materials, experimental systems and methods used in many studies. Here, indicate whether each material, system or method listed is relevant to your study. If you are not sure if a list item applies to your research, read the appropriate section before selecting a response.

Materials & experimental systems

n/a	Involved in the study	
[x]	[ ]	Antibodies
[x]	[ ]	Eukaryotic cell lines
[x]	[ ]	Palaeontology and archaeology
[x]	[ ]	Animals and other organisms
[x]	[ ]	Human research participants
[x]	[ ]	Clinical data
[x]	[ ]	Dual use research of concern

Methods

n/a	Involved in the study	
[x]	[ ]	ChIP-seq
[x]	[ ]	Flow cytometry
[x]	[ ]	MRI-based neuroimaging
